# Case Report: Bevacizumab combined with chemotherapy followed by PARP inhibitor maintenance therapy in *POLE*-mutated primary fallopian tube carcinoma: a case of precision treatment in a rare gynecologic malignancy

**DOI:** 10.3389/fmed.2026.1696996

**Published:** 2026-02-24

**Authors:** Donghai Cheng, Xueqin Huang, Peng Liu, Xiujie Liu, Wenyuan He

**Affiliations:** 1 People’s Liberation Army General Hospital of Southern Theatre Command, Guangzhou, China; 2 Nottingham Ningbo China Beacons of Excellence Research and Innovation Institute, University of Nottingham, Ningbo, China

**Keywords:** adenosquamous carcinoma, anti-angiogenic therapy, fallopian tube carcinoma, niraparib, *POLE* mutation

## Abstract

**Background:**

Primary fallopian tube carcinoma (PTC) is a rare epithelial malignancy of gynecologic origin. The adenosquamous carcinoma variant with *POLE* mutations is particularly uncommon, and current treatment strategies are limited by its elusive pathogenesis. Given similarities in biological behavior and clinical characteristics to ovarian cancer, PTC staging and management follow ovarian cancer guidelines. Previous studies suggest that anti-angiogenic therapy and poly (ADP-ribose) polymerase (PARP) inhibitors may be potential therapeutic options.

**Case presentation:**

We report a case of bilateral PTC exhibiting histologic heterogeneity and a *POLE* mutation, complicated by pelvic recurrence with bilateral lung metastases and sigmoid colon involvement. Partial response (PR) was achieved after six cycles of albumin-bound paclitaxel plus carboplatin (nab-TC) combined with bevacizumab (Bev), followed by maintenance therapy with the PARP inhibitor niraparib.

**Conclusion:**

This case demonstrates that Bev combined with chemotherapy may be an effective first-line regimen for this rare PTC variant. Maintenance therapy with a PARP inhibitor may prolong progression-free survival.

## 1 Introduction

Primary tubal carcinoma (PTC) is a rare gynecological malignancy that most commonly occurs in postmenopausal women between 50 and 60 years of age, with an incidence ranging from 0.14 to 1.8% among all female genital tract malignancies (1). Due to its histopathological resemblance to epithelial ovarian cancer, the treatment of PTC is largely guided by the standard management strategies for epithelial ovarian cancer.

The present case involves advanced primary bilateral tubal carcinoma with different histological subtypes and multiple organ metastases. Genetic testing identified a point mutation in the *POLE* gene. Such mutations can lead to ultra-mutation or a hypermutator phenotype in tumor cells, which may influence response to conventional chemotherapy. Concurrently, the resulting high tumor mutational burden can generate numerous neoantigens, thereby offering potential targets for precision immunotherapy (2–4).

This study reports a rare case of primary tubal carcinoma and reviews relevant domestic and international research advances. We further discuss the clinical characteristics and treatment strategies, with particular emphasis on the molecular features associated with *POLE* mutations, in order to provide insights for clinical diagnosis and treatment.

## 2 Patient information

**Figure 1:**
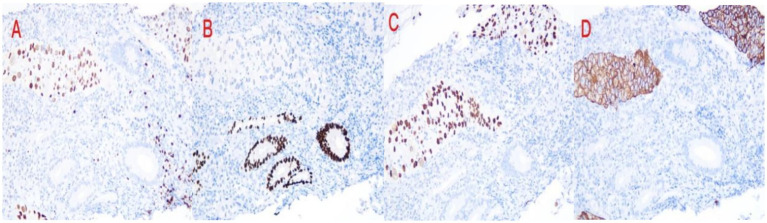
Partial immunohistochemical staining of sigmoid colon biopsy: **(A)** PAX-8(+), **(B)** CDX2(−), **(C)** P63(+), **(D)** CK5/6(+).

**Figure 2:**
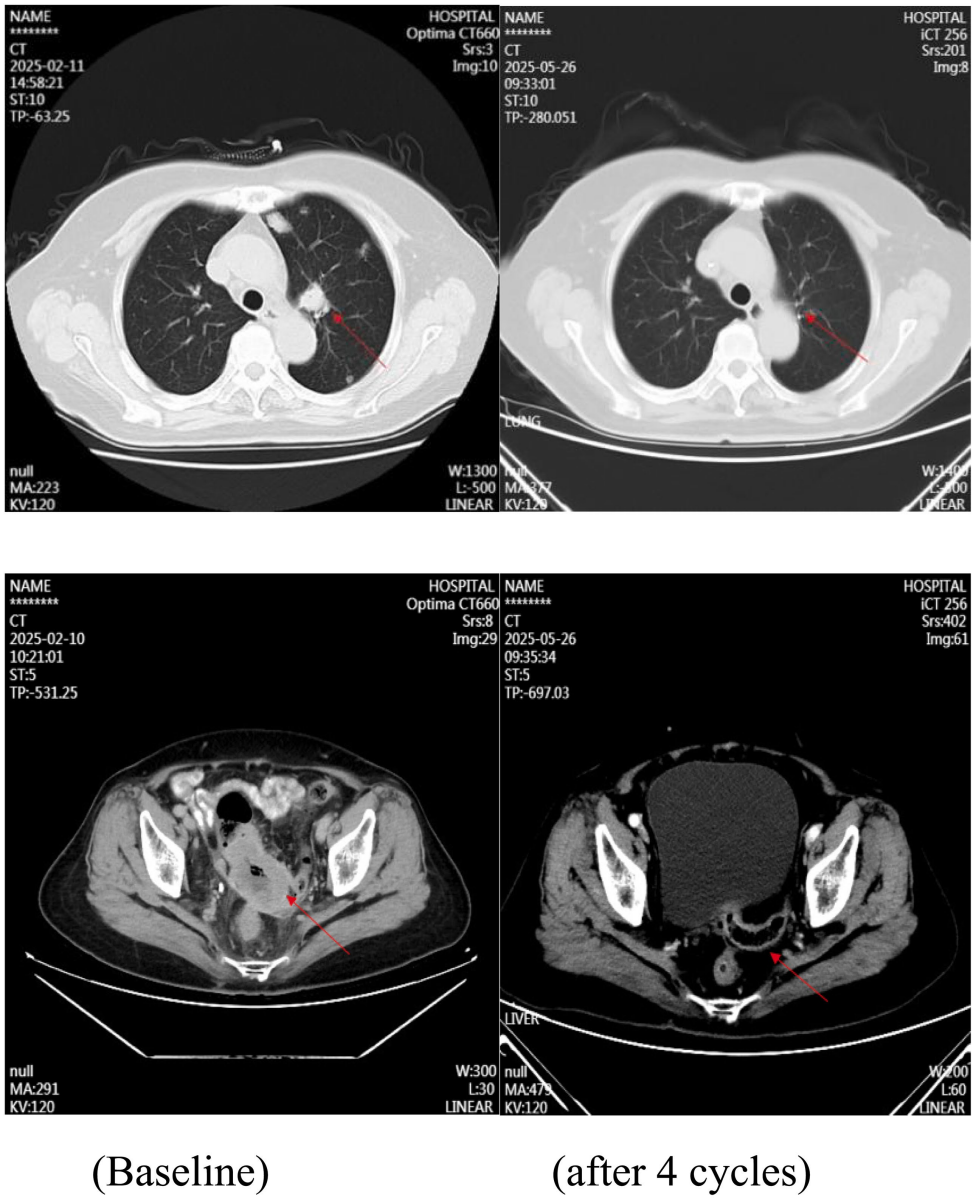
Pulmonary metastatic lesions and pelvic recurrent lesions before and after treatment.

The patient had previously undergone left salpingectomy for primary tubal carcinoma. Notably, there was no documented family history of gynecologic or related malignancies. Postoperative pathological examination of the left fallopian tube revealed atypical glandular epithelium arranged in glandular, cribriform, and papillary patterns within a fibrous stroma. Immunohistochemistry (IHC) results were as follows: ER (−), PR (−), WT-1 (−), PAX-8 (+), P53 (scattered positive, wild-type pattern), P16 (−), and Ki-67 approximately 30% positive, consistent with moderately differentiated adenocarcinoma. Pathological assessment of the right adnexal specimen showed atypical epithelial cells arranged in glandular, papillary, and nested patterns within a fibrous background. The tumor cells exhibited abundant lightly basophilic to eosinophilic cytoplasm, vesicular nuclei with prominent nucleoli in focal areas, and partial keratinization with keratin pearl formation. IHC staining results included: ER (−), PR (−); adenocarcinoma components were positive for CK7 and CK20; squamous cell carcinoma components were positive for CK5/6 and P63; CK19 (+), P16 (−), P53 (scattered positive, wild-type pattern), PAX-8 (+), WT-1 (−), and Ki-67 approximately 30% positive (Figure 1). These histomorphological and immunohistochemical features supported a diagnosis of moderately differentiated adenosquamous carcinoma. The patient declined adjuvant chemotherapy following surgery. Subsequently, the patient presented with unexplained hematochezia. Contrast-enhanced CT imaging demonstrated multiple pulmonary nodules, most exhibiting cavitation, along with pelvic masses, retroperitoneal lymph node enlargement, and suspected sigmoid colon invasion, suggestive of tumor recurrence or metastasis (Figure 2). Colonoscopy revealed an exophytic, cauliflower-like mass in the sigmoid colon with surface ulceration and active bleeding, resulting in luminal narrowing and obstruction. Histopathological examination of the colonic biopsy showed nests of atypical cells with abundant eosinophilic cytoplasm, ill-defined cell borders, enlarged oval nuclei, and conspicuous nucleoli. IHC results were as follows: ER (−), PR (−), pan-CK (+), CK7 (focally +), CK20 (−), CDX2 (−), P63 (+), CK5/6 (+), PAX-8 (+), Villin (−), GATA-3 (−), P53 (scattered positive, wild-type pattern), WT-1 (−), and Ki-67 approximately 60% positive (Figure 1). These findings confirmed the diagnosis of moderately differentiated squamous cell carcinoma.

To further characterize the molecular profile and identify potential therapeutic targets, next-generation sequencing (NGS) was performed. The results indicated HRD-negative status (score = 2), wild-type BRCA1/2, microsatellite stability (MSS), and pathogenic mutations in *POLE* (exon19 c.2053C>T, p.R685W; variant allele frequency 51.13%) and POLD1 (exon17 c.2136G>A, p.P712=; VAF 44.58%) (Table 1).

**Table 1:** Gene detection mutation map.

Gene	Reference sequence	Observed result	Location	VAF (%)
POLE	NM_006231.4	c.2053C>T p.R685W	Exon19	51.13
POLD1	NM_002691.4	c.2136G>A p.P712=	Exon17	44.58%

Based on existing literature and the molecular profiling results, the patient was treated with a paclitaxel/carboplatin regimen (nab-paclitaxel 260 mg/m^2^ + carboplatin AUC 5). Hematochezia improved after one cycle and resolved completely after two cycles, with radiographic evaluation indicating a partial response (PR). The patient then received four additional cycles of combination chemotherapy plus bevacizumab (800 mg), followed by maintenance therapy with niraparib (200 mg daily). The patient continues to be followed closely.

## 3 Discussion

Primary tubal carcinoma (PTC) is a rare gynecologic malignancy, most frequently exhibiting papillary serous or adenocarcinoma histology. The adenosquamous subtype with *POLE* mutations is exceptionally rare. This subtype combines biological features of both adenocarcinoma and squamous cell carcinoma, demonstrates aggressive behavior, and is associated with a poorer prognosis compared to pure adenocarcinoma (5, 6). To our knowledge, this represents the first reported case of primary tubal carcinoma exhibiting distinct bilateral pathological heterogeneity—adenocarcinoma on the left and adenosquamous carcinoma on the right—accompanied by recurrent metastatic disease. Meanwhile, the characteristic of *POLE* signature is currently being analysed and will be shared in a near future. Given the favorable response observed with TC chemotherapy plus bevacizumab followed by niraparib maintenance, we discuss the biological heterogeneity, clinical implications of the *POLE* mutation, therapeutic strategies, and future directions.

### 3.1 Biological implications of tumor heterogeneity

Tumor heterogeneity is a key biological characteristic of malignant tumors, occurring at genomic, transcriptomic, and phenotypic levels. In this case, the primary lesion was diagnosed as fallopian tube adenosquamous carcinoma, while the recurrence and metastasis presented as pure squamous cell carcinoma, illustrating dynamic phenotypic evolution. Although the role of the *POLE* mutation in this transition remains unclear, current evidence suggests that *POLE*-mutant tumors display genomic instability, which may facilitate clonal evolution and drive progression toward a more aggressive squamous phenotype.

Recent studies have highlighted the complex interplay between the tumor microenvironment and tumor heterogeneity (7). The tumor microenvironment, consisting of various immune cells, stromal cells, and extracellular matrix components, can significantly influence tumor evolution. For instance, immune cells in the microenvironment can either suppress or promote tumor growth and metastasis depending on their activation state and the cytokines they secrete. In the context of POLE-mutant tumors, the immune-rich microenvironment with significant tumor-infiltrating lymphocytes may play a dual role. On one hand, it could potentially contribute to the immune surveillance and control of tumor growth; on the other hand, it might also drive the selection of more aggressive tumor clones that can evade immune recognition, leading to the observed phenotypic evolution from adenosquamous to pure squamous cell carcinoma.

### 3.2 Molecular features and clinical significance of POLE mutation

*POLE*-mutant tumors are typically characterized by an ultrahigh tumor mutational burden (TMB), microsatellite stability (MSS), and an immune-rich microenvironment with significant tumor-infiltrating lymphocytes. Clinical studies have shown that patients with *POLE*-mutant solid tumors respond more favorably to immune checkpoint inhibitors compared to those with *POLE*-wild-type tumors (8, 9). However, most reported *POLE* mutations occur within the exonuclease domain (exons 9–14), whereas the mutations in this case were located in non-exonuclease domains (exons 17 and 19). The clinical significance of such variants remains uncertain. Systemic host factors also play a crucial role in determining the response to treatment in patients with POLE-mutant tumors. Pre-treatment physiological status, such as overall health, nutritional status, and the presence of comorbidities, can significantly influence immune recovery and treatment tolerance. For example, patients with poor nutritional status may have a weakened immune system, which could limit the effectiveness of immune-based therapies. Additionally, psychological factors, including stress, anxiety, and depression, can impact the immune system through the hypothalamic-pituitary-adrenal axis and the sympathetic nervous system, potentially affecting treatment outcomes.

Furthermore, given the patient’s BRCA wild-type status, HRD negativity, and MSS profile, limited benefit was expected from PARP inhibitors or immunotherapy alone. Based on these findings, first-line therapy with TC plus bevacizumab was initiated, resulting in a partial response after six cycles, confirming the regimen’s efficacy.

### 3.3 Rationale and challenges of PARP inhibitor maintenance

The phase III PRIMA and PRIME trials demonstrated that niraparib significantly improves progression-free survival (PFS) across all subgroups of patients with advanced ovarian cancer. Notably, patients with BRCA wild-type and HRD-negative disease derived a median PFS benefit of 2.7 months (10, 11). However, these landmark trials primarily included patients with high-grade serous carcinoma, and the efficacy of PARP inhibitors in rare histologic subtypes such as tubal adenosquamous carcinoma—especially with concurrent *POLE* mutations—remains unclear. Mechanistically, *POLE* mutations induce genomic instability, and synergistic activity between PARP inhibition and immunotherapy may promote synthetic lethality (12). Nevertheless, the molecular mechanisms of *POLE* mutations are multifaceted, and the impact of specific mutation loci on treatment response warrants further clinical validation. Recent insights into the tumor microenvironment and immunotherapy interactions have shown that microbial-immune dynamics can shape treatment responsiveness (13). The gut microbiota, for example, can modulate the immune system and influence the efficacy of immune checkpoint inhibitors. It is possible that the microbial composition in patients with POLE-mutant tumors may interact with the tumor cells and the immune cells in the tumor microenvironment, affecting the response to PARP inhibitors and immunotherapy.

After multidisciplinary discussion and informed consent, niraparib maintenance therapy was initiated. At the most recent follow-up (4 weeks post-chemotherapy), the patient exhibited no radiologic evidence of disease progression and reported no significant treatment-related toxicities. However, long-term follow-up is essential to assess the durability of the response and to monitor for potential late-onset toxicities.

### 3.4 Future therapeutic directions

Emerging evidence indicates that *POLE*-mutant tumors demonstrate objective response rates of 30–50% to PD-1/PD-L1 inhibitors, regardless of microsatellite status (8, 14), supporting the use of immune checkpoint blockade, neoantigen-based vaccines, and T-cell therapies. Combination strategies may further improve outcomes: PARP inhibitors may synergize with immunotherapy through synthetic lethality and immunomodulatory effects. In the TOPACIO trial, the combination of niraparib and pembrolizumab achieved a disease control rate of 78% in *POLE*-mutated ovarian cancer (15). Additionally, agents targeting DNA damage response pathways have demonstrated selective lethality in *POLE*-mutant preclinical models, with several regimens currently under evaluation in phase I trials (16). However, the hypermutator phenotype may accelerate clonal evolution and therapeutic resistance. Combined inhibition of ATR/WEE1 signaling or the incorporation of epigenetic modulators may help overcome resistance. Epigenetic changes, such as DNA methylation and histone modifications, can influence gene expression and tumor cell behavior. Modulating these epigenetic processes may help to reverse drug resistance and improve treatment efficacy (17). Overall, the management of *POLE*-mutant tumors is evolving from empirical chemotherapy toward molecularly guided precision therapy. Future studies should further elucidate the interplay between *POLE* mutations, co-occurring driver alterations, and immune microenvironment remodeling to inform the development of more effective treatment strategies.

## 4 Conclusion

This case highlights the distinct clinical, molecular, and therapeutic challenges posed by primary tubal adenosquamous carcinoma with *POLE* mutation. Bilateral pathological heterogeneity and aggressive recurrence emphasize the necessity for precision-based, individualized treatment. The observed clinical benefit from TC chemotherapy plus bevacizumab followed by niraparib maintenance suggests a potential survival advantage, though validation in larger cohorts with longer follow-up is needed. Future advances in multidisciplinary management, coupled with comprehensive molecular profiling—including BRCA, HRD, MSI, and *POLE* status—are expected to enhance the integration of precision medicine and novel therapeutics, ultimately improving outcomes for patients with rare gynecologic malignancies.

## Data Availability

The datasets presented in this study can be found in online repositories. The names of the repository/repositories and accession number(s) can be found in the article/supplementary material.
